# How to Culture, Record and Stimulate Neuronal Networks on Micro-electrode Arrays (MEAs)

**DOI:** 10.3791/2056

**Published:** 2010-05-30

**Authors:** Chadwick M. Hales, John D. Rolston, Steve M. Potter

**Affiliations:** Department of Neurology, Emory University; Coulter Department of Biomedical Engineering, Laboratory for Neuroengineering, Georgia Institute of Technology and Emory, University School of Medicine; Emory University

## Abstract

For the last century, many neuroscientists around the world have dedicated their lives to understanding how neuronal networks work and why they stop working in various diseases. Studies have included neuropathological observation, fluorescent microscopy with genetic labeling, and intracellular recording in both dissociated neurons and slice preparations. This protocol discusses another technology, which involves growing dissociated neuronal cultures on micro-electrode arrays (also called multi-electrode arrays, MEAs).

There are multiple advantages to using this system over other technologies. Dissociated neuronal cultures on MEAs provide a simplified model in which network activity can be manipulated with electrical stimulation sequences through the array's multiple electrodes. Because the network is small, the impact of stimulation is limited to observable areas, which is not the case in intact preparations. The cells grow in a monolayer making changes in morphology easy to monitor with various imaging techniques. Finally, cultures on MEAs can survive for over a year in vitro which removes any clear time limitations inherent with other culturing techniques.^1^

Our lab and others around the globe are utilizing this technology to ask important questions about neuronal networks. The purpose of this protocol is to provide the necessary information for setting up, caring for, recording from and electrically stimulating cultures on MEAs. *In vitro* networks provide a means for asking physiologically relevant questions at the network and cellular levels leading to a better understanding of brain function and dysfunction.

**Figure:**
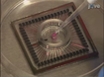


## Protocol

### A. Introduction

There are billions of neurons in the human brain. Each of these neurons can have hundreds to thousands of connections often times with many different cells. These connections harbor the signals that allow us to walk, play a piano, ride a bicycle, laugh, cry, and remember. For the last century, many neuroscientists around the world have dedicated their lives to understanding how neuronal networks work and why they stop working in various diseases.

There are many tools one can use when taking on this seemingly insurmountable task. Initial studies were focused on neuropathological observation of how neural circuits are organized in the brains of humans and other animals. The advent of fluorescence microscopy and genetic labeling expanded the scope of these structural studies exploring cellular composition down to the protein and DNA level.

But these experiments did not address the dynamic function of the brain, only the static arrangement. For understanding ongoing neural activity, popular techniques generally focus on examining electrophysiological activity with intracellular recording. Single neurons of dissociated cultures provide a useful reductionist model, however this technique is limited by short time intervals for recording from single cells. This model also provides limited information about other cells in the network. Brain slices from rodents provide more of a realistic model where cortical architecture is maintained. But such slices, even when cultured, have a limited life span and can be technically challenging to keep alive while retaining cytoarchitecture^2^.

Another technology involves growing dissociated neuronal cultures on micro-electrode arrays (also called multi-electrode arrays, MEAs). Neurons are plated onto MEAs which have microelectrodes embedded in the bottom of the dish. Over the course of three weeks, these cultures form networks of neurons complete with axons, dendrites and hundreds if not thousands of synaptic connections. There are multiple advantages to using this system over other technologies. Dissociated neuronal cultures on MEAs provide a simplified model with which to work (on the order of a single cortical column rather than an intact brain). The cells grow in a monolayer making changes in morphology easy to monitor with various imaging techniques. Network activity can be manipulated with electrical stimulation sequences through the array's multiple electrodes. Because the network is small, the impact of stimulation is limited to observable areas, which is not the case in intact preparations. Finally, cultures on MEAs can survive for over a year *in vitro* which removes any clear time limitations inherent with other culturing techniques.^1^ Therefore, cultures on MEAs represent an ideal model for studying neuronal connectivity.

Our lab and others around the globe are utilizing this technology to ask important questions about neuronal networks. Current neuronal processes being studied with this model include network dynamics, development, learning and memory, synaptic plasticity, excitotoxicity, ischemia and neurodegeneration.^3-10^ Findings from these studies could have significant implications for establishing better treatments for human diseases like congenital malformations, epilepsy, stroke and Alzheimer's disease. The purpose of this protocol is to provide the necessary information for setting up, caring for, recording from and stimulating cultures on MEAs. The ultimate goal is to provide a means for researchers to ask physiologically relevant questions at the cellular and network levels that may perhaps lead to a better understanding of brain function and dysfunction and ultimately the treatment of diseases that affect our neurons and how they communicate.

The following protocol represents over 10 years of experience from our lab in culturing, recording from and stimulating neuronal networks on micro-electrode arrays. Each step has been optimized so as to lead to the development of long surviving healthy neuronal networks. References are provided where available while some optimal settings we determined empirically. Prior to initiating the protocol, obtain equipment and supplies and prepare solutions as listed in the section entitled 'Materials.' The section on 'Brain Dissection' will be briefly discussed, but not demonstrated in the accompanying video as other Journal of Visualized Experiment articles cover this topic^11^. 

### B. Brain Dissection

1. Whole brains are extracted from embryonic day 18 (E18) rats. Timed-pregnant female rats are anesthetized with inhaled isoflurane and decapitated. Embryos are removed and dissected according to the National Research Council's Guide for the care and use of laboratory animals using a protocol approved by the Emory University IACUC.
2. While utilizing aseptic technique, the embryonic brains are removed and placed in cold Hank's Balanced Salts solution (HBSS) in a 100 mm sterile Petri dish. The remainder of the dissection protocol occurs under a dissecting microscope in a laminar flow hood to minimize risk for contamination.
3. The rat brains are transferred one at a time to a second sterile Petri dish with HBSS for dissection. While submerged in HBSS, the brainstem, thalamus and cerebellum are cut away and the hemispheres are bisected.
4. The olfactory tubercle is utilized as a handle for securing the hemisphere while gently removing the meninges.
5. The cortex is then peeled away from the underlying hippocampus and striatum and stored in a sterile 15 ml conical tube in hibernate solution on ice.^12^ Since there are usually multiple embryos, this procedure is then repeated depending on the desired number of MEAs to be plated and the desired cell density. Cortices are combined for the cell dissociation process as discussed below. Cortices may be stored at 4°C in hibernate solution for up to 24 hours prior to dissociation with only minimal loss of cell viability. As an alternative to utilizing tissue dissected in-house, dissected brain tissue can be purchase from Brain Bits (www.brainbitsllc.com).

### C. MEA preparation and cortical dissociation

1. The MEAs are obtained from ALA Scientific (www.alascience.com), a vendor for the manufacturer, Multi-Channel Systems. There are several different configurations to the MEAs with variations in electrode orientation and spacing, presence or absence of an internal ground electrode, presence or absence of a glass ring, and the size of the glass ring. For our basic experiments we use the standard micro-electrode array containing 60 electrodes in an 8 by 8 grid arrangement with a small glass ring. The titanium nitride electrode diameter is 30 μm and the distance between electrode centers is 200 μm. One of the electrodes is larger and functions as the ground or internal reference electrode. When handling MEAs, it is vitally important that no solid objects (e.g., pipette tips, paper tissues, or rubber policemen) ever touch the inside of the dish as this can damage the electrodes. More detailed information about MEAs and handling can be found in the MEA user manual (www.multichannelsystems.com/products-mea/microelectrode-arrays.html).
2. On the evening prior to the cell preparation, the MEAs are rinsed with de-ionized water and then allowed to soak in 70% ethanol for 15 minutes. The dishes are then placed in a laminar flow hood with the UV light on overnight. For handling purposes, each MEA is placed in a standard 100 mm sterile polystyrene Petri dish with dates and labeling placed on the Petri dish. Of note, other sterilization techniques including autoclaving and utilizing water at 90°C are mentioned in the MEA users manual. However, we have empirically found that autoclaving seems to decrease the number of times that an MEA can be used. If you are reusing MEAs that currently have cultures, then the organic matter must be removed. 300-400 μl of 0.25% trypsin in PBS is incubated on the MEA at 35-37°C for 20 minutes. The MEA is rinsed with de-ionized water and then inspected with a light microscope. If cellular matter still remains, then the trypsin step is repeated until clean. If the dish is clean, then proceed with the sterilization step as above. In general, MEAs can be reused multiple times with the appropriate care.
3. Also on the evening prior to plating, the MEA lids are prepared and autoclaved. The lids are custom made but can also be purchased from ALA-Scientific. They are made of polytetrafluoroethylene Teflon and have a clear membrane (fluorinated ethylene-propylene Teflon) stretched across the top. The membrane allows the diffusion of gases but limits the diffusion of water thereby virtually eliminating the need for a humidified atmosphere and preventing the deleterious effects of evaporation and hyperosmolarity.^1^
4. Once the dissection above is complete, MEA preparation is continued by placing 100 μl of polyethyleneimine (PEI) solution into the center of each MEA.^13^ In our hands, PEI solution provides less clustering of cells in the MEA than using polylysine. The dish is allowed to sit at room temperature in the laminar flow hood for 30 minutes with lids lightly resting on the MEAs to prevent evaporation. As a reminder, any work with the open MEA or brain tissue should take place in a standard cell culture laminar flow hood to minimize the risk of contamination.
5. The MEA is then rinsed 3 times with 1-2 ml of sterile de-ionized water. The ideal laminar flow hood set-up will include an aspirator apparatus for removing liquid from dishes. A sterile 200μl pipette tip can be attached to the aspirator tubing. Do not touch the surface of the MEA while aspirating as this can damage the electrodes. After the final rinse, the MEA is allowed to dry for at least 30 minutes in the laminar flow hood.
6. During this drying period, the neuronal dissociation process is started by placing the cortices into 2 ml of papain solution in a 15 ml sterile conical plastic vial.^1^ 50 μl of DNAse is added to the mixture. The vial is placed into a water bath at 35-37°C and incubated for approximately 20 minutes. Gently tap the solution every 5 minutes for mixing being careful to avoid introducing bubbles. The cortices should begin to take on a fuzzy appearance as the digestion proceeds.
7. The papain solution is then carefully removed by pipetting leaving the cortices in the 15ml conical vial. 2 ml of cell medium is added to the cortices, allowed to incubate for 2-3 minutes and then gently removed. This is to wash out any residual papain, which will also be inhibited by the serum in the medium. Another 2ml of cell medium is added to the cortices and this sample is then vortexed on high speed for 5-10 seconds. There should be a cloudy solution in the bottom of the 15ml conical vial and no remaining brain pieces. Alternatively, trituration with 1 ml of medium by 3 times using a P-1000 hand-held pipette can be utilized for dissociating the tissue.^1^ For proper trituration, each pipette stroke should take one second.
8. The solution is then allowed to pass gently through a sterile 40μm cell strainer via gravity. Slowly add the cell solution one drop at a time into the strainer with a P-1000. A 35 mm sterile Petri dish is utilized for collecting the strained cell solution.
9. The cell suspension is then transferred back to a 15 ml conical vial and cell medium is added to a final volume of 4.0 ml.^14^
10. 500 μl of a 5% w/v BSA solution is then carefully layered into the bottom of the 15ml conical vial with a P-1000.^14^ This will displace the cell-containing solution upwards in the vial.
11. The cell solution with BSA layered in the bottom is then centrifuged for 6 minutes at 200 x g to remove small debris and potentially toxic intracellular contents.^14^
12. During the cell centrifugation step, the MEAs are visually assessed to ensure they are completely dry. 20 μl of laminin solution are then carefully placed into the center of the MEA ensuring that all of the electrodes are covered.^1^ Avoid introducing air bubbles into this solution since that can lead to inadequate preparation of the electrode surface. If the MEA is not completely dry, then the drop of laminin will spread across the dish potentially making it more difficult to adequately localize the cells over the electrodes when plating. This step is very delicate and has the potential to lead to MEA surface damage with an unsteady hand or lapse in attention.
13. The Teflon lids are placed onto the MEA at this point to prevent evaporation of the laminin. Placement of the lid is also delicate. A lid should only be placed or removed when the MEA is on a completely flat surface. Otherwise, extra forces exerted by an uneven surface may crack the glass bottom of the array rendering it unusable. The dish is then placed into the incubator for 20 minutes.
14. While the laminin is binding to the dish, the 15 ml conical vial containing the cells is removed from the centrifuge and transferred back to the laminar flow hood. A pellet should be visible in the bottom of the vial. The supernatant is carefully removed with a sterile 5ml plastic serological pipette. Save the supernatant in case the centrifugation process was incomplete. The pellet is then resuspended gently either by trituration with a P-1000 or a quick vortex in 300-500 μl of cell medium depending on the expected yield. 10 μl of cell suspension is removed and placed on a hemacytometer to determine cell concentration. If the yield is low, analyze the supernatant under the microscope to determine if the centrifugation step was incomplete. Repeat centrifugation may be necessary. If the yield is high, then a dilution may be necessary for more accurate counting. Cell plating density can range from 5,000 to 300,000 cells per MEA. Calculate a dilution so that the desired number of cells for plating is in a final volume of 15-20 μl. We typically use a final concentration of 1000 to 3000 cells per μl. One pair of cortices will typically yield between 10 and 15 million cells. This number may be slightly lower if trituration is utilized.
15. By this time, the laminin incubation on the MEA should be complete. The Teflon lid is removed from the MEA and most of the laminin drop is vacuum aspirated or pipetted away. Then 15-20 μl of the desired cell suspension is immediately pipetted onto the residual wet area of laminin. Be careful to avoid introducing air bubbles into this small volume of cell suspension as air bubbles can prevent cells from evenly covering all of the electrodes. The lid is gently replaced and the MEA is carefully placed back in the incubator for 30 minutes. The dish is then inspected under the light microscope to ensure that the cells have adhered and cover all of the electrodes. If all of the electrodes are not covered, then more cells can be added and the MEA is put back in the incubator with lid on to allow these new cells to adhere. If a tube of cell suspension is used more than a few minutes after sitting undisturbed, be sure to gently swirl it to resuspend settled cells. Once adequate electrode coverage is achieved, then the dish is slowly flooded with 1ml of warm (35-37°C) cell medium. The Teflon lid is replaced and the dish is placed back in the incubator. The laminar flow hood can quickly dry the small laminin volumes and cell suspension volumes once on the MEA so take care to keep them sealed with the Teflon lids. The ideal incubator environment for the sealed MEAs is 5% CO_2_, 9% O_2_ and 65% humidity at 35-37°C. Low oxygen tension is important for long-term cultures, reducing oxidative damage.^12^ Maintaining 65% humidity reduces evaporation through the Teflon membrane (compared to no humidity control) and allows MEA electronics to be used in the incubator without damage from water condensation (as with a normal incubator humidified to saturation).

### D. Changing cell medium and caring for dissociated cultures on MEAs

1. On the day after cell plating, inspect the MEA under the light microscope. If the color of the medium is still peach to red in color and there is a limited amount of cellular debris and cell death, then the first medium change can be delayed for another day. However, if the medium is starting to appear more orange or yellow in color and/or there is a large amount of cellular debris and cell death, then change the medium. All medium changes should occur in a standard cell culture laminar flow hood.
2. The first medium change consists of removing the MEA lid, aspirating away all of the medium in the dish, replacing the amount removed with fresh cell medium, and then replacing the lid. Replace the entire amount of the cell medium on the first medium change to remove any remaining cellular debris from the plating process. Subsequent medium changes should only aspirate away approximately half of the medium followed by replacing the amount removed with fresh medium. This allows some of the growth factors that have been secreted into the cell medium to remain with the cells maintaining a more optimal growing environment. If the underside of the lid is contaminated during the medium changing process or if the lid shows signs of damage or loose fitting, then utilize a new autoclaved lid. Cell medium can be stored in the incubator in a sterile container with a custom modified lid (that has a Teflon (fluorinated ethylene-propylene) membrane to allow for gaseous interchange). Cell medium can also be stored at 4°C but warm and equilibrate this in the incubator prior to changing medium.
3. Remove and replace approximately half of the medium in a dish about twice a week. Some cell preparations may lead to more rapid color changes in the medium causing a need for more frequent changes. If medium is noted to be yellow, then change the entire amount of medium. Rapid transition to yellow can also be the sign of contamination so inspect the dish under the light microscope for visual signs of infection.
4. Depending on experimentation conditions and sterile technique, cultures that have been carefully cared for can survive for more than a year.^1^

### E. Recording from MEAs

1. Each MEA has 59 recording electrodes and one internal ground electrode. There are several commercial recording systems available as well as custom made versions. Vendors include Multi-Channel Systems, Tucker Davis Technologies, Axion Biosystems, Alpha Med Scientific MED64, Plexon, Ayanda Biosystems and BioLogic. Our laboratory currently uses a commercial setup from Multi-Channel Systems, a custom designed Windows based system called NeuroRighter^15^, and a custom designed Linux based system called MeaBench^16^. Each of these systems is able to record from MEAs (Multi-Channel Systems) using a Multi-Channel Systems preamplifier. A commercial setup from Tucker Davis Technologies is also in use. This protocol will demonstrate recording from MEAs with NeuroRighter. NeuroRighter software is open-source and available for free download along with the hardware designs on NeuroRighter Google groups. (http://sites.google.com/site/neurorighter/)
2. Cultures on MEAs take 2-3 weeks to reach a steady state of activity.^17,18^ This activity includes spontaneous action potentials and culture-wide bursting (a barrage of action potentials that spreads synaptically across a culture). The nature of the experiment however will determine the ideal number of days *in vitro *before recording.
3. Neuronal activity is dependent on ideal environmental conditions including temperature and pH.^1^ As a result, our recording takes place in the incubator as described above. If short experiments (<30 minutes) are designed, there is also a commercially available heating module that can be connected to the MCS preamplifier for maintaining the ideal MEA temperature.
4. To start recording, a MEA (still in its Petri dish) is gently transferred from the storage incubator to the recording incubator. Keep the bottom of the dish parallel to the ground. Avoid quick movements with the MEA or jarring the MEA as these can detach the culture from the substrate. The MEA is then removed from the Petri dish and placed in the recording preamplifier. Match the internal ground electrode in the dishes we are currently demonstrating with the ground electrode on the preamplifier (channel CR15 for the MCS preamplifier). Wipe the contacts around the perimeter of the MEA using a tissue or cotton swab moistened with 70% ethanol to remove fingerprints or other debris that may hinder a good connection. After ensuring that the MEA is correctly seated, the preamplifier lid is then lowered and latched into place. The contacts on the preamplifier lid should rest firmly on the electrode contact pads on the MEA. Visually inspect contact alignment and adjust with a cotton swab if necessary. Place the preamp onto the Peltier device in the incubator. This consists of two copper plates with a Peltier thermoelectric heat pump sandwiched between them. We run this at about 5V, to remove some of the heat produced by the preamp circuitry. This prevents condensation from forming on the inside of the Teflon membrane lid. Any condensation would indicate harm the culture by increase in osmolarity of the medium in contact with the cells, due to evaporation. By ensuring the that preamp and the medium are a degree or two below the incubator air temperature, changes in osmolarity are minimized.
5. Turn on the power supply to NeuroRighter and open the software on the workstation. Adjust NeuroRighter settings to *in vitro* with appropriate software/hardware configurations also in place. Select the number of electrodes in the software. In order to reduce the amount of background noise, a digital band-pass filter can be enabled. Select a data file with the record switch on if data is to be saved for later analysis. Activate the start button once all appropriate settings are chosen.
6. The screen should show running traces of activity and background noise. Occasional action potentials and dish wide bursts of activity should be visible. The section below on troubleshooting recording from MEAs will discuss several common issues if expected activity is not visualized.
7. The screen view can be changed to spike detection mode ('Spk Wfms' tab) where action potentials are displayed statically as they occur in 3ms time windows. For a given channel, real extracellular action potentials should last approximately 1ms and should fire repeatedly in the same direction (positive or negative). Occasionally, two or more neurons can be visible on the same electrode with different polarities. Spikes with a short time course and variable polarity are likely artifact. Figure 1 shows a raster plot of spike data.
8. The screen view can be changed to local field potentials (LFPs) for a look at localizing the origin of bursting activity. This type of setting can be more useful when recording *in vivo* since monolayer cultures have weak LFPs. Of note, some preamplifiers (including one we use from Multi-Channel Systems) have high-pass filters at 10 Hz which will attenuate lower frequency LFP rhythms.

### F. Stimulating neuronal networks on MEAs

1. The same 59 recording electrodes on a MEA can also be utilized for stimulating the culture. The nature of the experiment will determine the extent of stimulation. NeuroRighter provides flexibility for stimulation experimental design since it is open source code.^15^
2. The challenge with stimulating a culture is that the stimulus creates an artifact that can last several milliseconds. This artifact can be large enough to trigger spike detection and obscure stimulus responses. NeuroRighter utilizes the SALPA algorithm for minimizing and in some cases essentially eliminating the stimulus artifact.^19^
3. To stimulate the neuronal network on the MEA, train the SALPA algorithm under the 'Recording settings' tab to limit the stimulation artifact. Activate SALPA in the 'Online Settings' tab. Select a file name and click the Start button as previously to begin recording. Then click on the 'Stim' tab. There are many choices for setting up stimulation on this page.
4. A simple experiment is to stimulate one electrode and watch the responsiveness of the dish using the 'Open loop' section. Select the rate, voltage, phase width and channel number. Press the start button in the open loop section. Responses to this stimulation can be visualized on the main 'Spikes' tab and the 'Spk Wfms' tab and analyzed in the data files.
5. In real time, bursts can be visualized in a time-locked manner to a one hertz stimulus of 0.5V across multiple electrodes. Figure 2 shows a raster plot of a post-stimulus response. Prior observations have demonstrated that there are two different types of stimulus-evoked activity: directly evoked action potentials (dAPs) and synaptically evoked action potentials (sAPs).^20^
6. Neurons in culture have spontaneous bursts of activity. For some experiments, this type of bursting can interfere with attempting to control the network dynamics. Interestingly, distributed stimulation at 1-2 Hz per electrode can start to suppress bursting activity in a neuronal network on a MEA.^21^

### G. Trouble-shooting recording from MEAs

1. No action potentials or bursting present in the MEA when the software is turned on:
  1. Is the culture old enough? Action potentials should be visible toward the end of the first week *in vitro*. Bursting occurs around two to three weeks *in vitro*.
  2. Is the hardware receiving power? The NeuroRighter system utilizes two 6V lead-acid batteries to power the hardware. The power switch needs to be in the on position and the batteries charged.
  3. Is the culture alive? At times this can be challenging to determine especially in dense or older culture because of glial cell overgrowth. However, one can look for healthy appearing neurons (shiny, elliptical shaped cell bodies under phase contrast microscopy) and intact (not fragmented) cell processes. There may also be a problem with the culture if there is evidence for visible contamination or rapidly degrading medium (becoming acidic after only 1 to 2 days between medium changes).
  4. What is the electrode impedance? Over multiple uses, the micro-electrodes or the insulation of a MEA can degrade. NeuroRighter has the capability to assess the electrode impedance.^15^ Normal impedance readings around 1kHz should range between 10,000 and 100,000 Ohms. Higher impedances suggest broken leads or electrodes and readings less than 10,000 Ohms suggest leaky insulation.
  5. Is the preamplifier connected to the hardware board? The preamplifier should have an output cable that connects to the hardware board. The preamplifier also has 4 small stimulator boards that also connect to the main hardware board and are important for stimulation only.
  6. Have the NeuroRighter software settings been changed? Hardware configuration settings in the software must be correct for recording to work. Installing updated versions and multiple users changing settings can cause this.
  7. Is the MEA at 35-37°C? Cooler temperatures can lead to a reduction in spontaneous activity in cortical networks. This is usually not an issue since recording occurs within the climate-controlled incubator, however one must allow for temperature equilibration if the MEA has been outside of the incubator for more than a few seconds.
  8. Has the medium been changed in the past few hours? We have observed the cultures often stop firing for several hours after feeding. Therefore, it is best to conduct experiments before the feeding.
2. Electrode with excessive background noise saturating the recording window:
  1. Is the preamplifier pin contact with the electrode on the MEA sufficient? This problem can be caused by a misaligned pin on the preamplifier lid, a small piece of trash, a drop of medium, a degraded electrode surface, or an overlapping clear membrane from the lid. Inspecting the contact for these issues is the first step. Adjusting the pin with a cotton swab soaked in 70% ethanol can sometimes improve the contact especially if there was a drop of medium that needed cleaning away. The signal may appear worse until the ethanol evaporates.
  2. Does the electrode appear to be damaged? Visualization under the light microscope can sometimes reveal cracked or pitted electrode insulation leading to this type of artifact.
  3. Are the contact pads worn or scratched? Sometimes there may be several electrode contacts on the MEA that are difficult to optimize. This is more common after multiple MEA uses and platings. A gold wire impregnated rubber spacer conducts only in the direction of its 0.5mm thickness and can improve preamplifier pin contact with the MEA. This material is available from Fujipoly and can be cute to fit the MEA and lid.
3. Entire MEA shows excessive background noise:
  1. Is the MEA in the correct orientation so as to allow the ground electrode on the MEA to contact the ground pin on the preamplifier? If the MEA is in the correct orientation then ensuring there are no ground contact issues (2 a-c above) is also important to consider. Is channel CR15 grounded with a short wire to the preamp chassis? Using the jumper to ground it through internal 30 kOhm resistors may result in excess noise.
  2. Is there interference from surrounding lights, power sources or other pieces of equipment? One of the most common forms of interference is 60 Hz from lights and equipment. The recording system must be properly grounded. Shielding exposed cables can also be helpful at reducing background interference.

### Representative Results


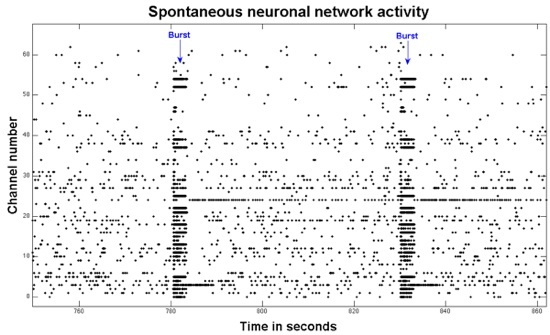
 **Figure 1.**This is a raster plot of 2 minutes of spontaneous activity from a neuronal network. Each black dot represents an action potential. Two bursts of activity are indicated with the blue arrows. The response over multiple electrodes is a function of the network connectivity.


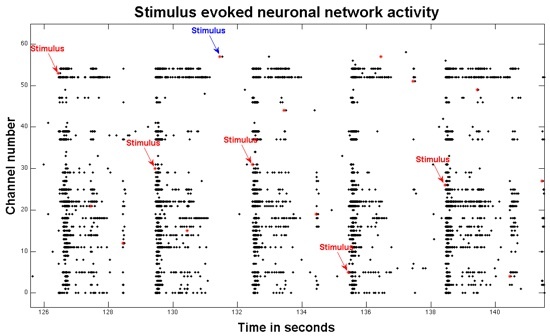
 **Figure 2.**Here is a raster plot of 16 seconds of stimulus-evoked activity. Red stars represent stimuli. The red arrows show five stimuli that successfully induced bursts of synaptic activity across multiple electrodes. Occasionally, a stimulus does not yield a burst, here at the blue arrow.

## Disclosures

CMH has nothing to disclose.<br /> JDR and SMP are paid consultants for Axion Biosystems.<br /> The submission fees and video processing fees for this article and video were graciously sponsored by Multi-Channel Systems.

## Discussion

This protocol shows instructions on how to culture and maintain neuronal networks on micro-electrode arrays as well as an introduction to recording from and stimulating neuronal networks. Some of the more common problems when recording from MEAs were also discussed in the MEA troubleshooting section. There are occasional variations in the protocol as discussed throughout and oftentimes these changes are utilized to optimize specific conditions required by certain experimental designs.

Although the recording and stimulating system presented here is comprised of our custom designed NeuroRighter^15^ with the MCS preamp, there are multiple other systems that can provide an interface with the neuronal networks. Choosing a particular setup will depend on the specific experimental needs.

Simplistic recording and stimulation examples were provided in this protocol however these cultures on MEAs can be used to provide insight into complex questions about synaptic activity and neuronal network connectivity. As mentioned above, ongoing work is utilizing this technology to study processes like cellular plasticity, development, excitotoxicity and neurodegeneration.^6-10^ For example, a recent publication from our laboratory showed reproducible goal directed learning *in vitro* with real-time closed loop stimulation in response to current neuronal network activity^3^.

Although MEA cultures are often finicky and delicate, thanks to their simplicity and accessibility (compared to intact animals), they provide a powerful model for studying neuronal activity at a network level.
